# The influence of suit size on performance in ski jumping. Part II: field measurements

**DOI:** 10.3389/fspor.2026.1693723

**Published:** 2026-02-13

**Authors:** Ola Elfmark, Sören Müller, Mario Kürschner, Veronica Bessone, Piotr Krężałek, Mikko Virmavirta

**Affiliations:** 1 Centre for Elite Sport Research, Department of Neuromedicine and Movement Science, Norwegian University of Science and Technology, Trondheim, Norway; 2 International Ski and Snowboard Federation, Oberhofen am Thunersee, Switzerland; 3 Department of Strength, Power and Technical Sports, Institute for Applied Training Science, Leipzig, Germany; 4 Department of Biomechanics, Institute of Biomedical Sciences, University of Physical Culture, Krakow, Poland; 5 Faculty of Sport and Health Sciences, Biology of Physical Activity, University of Jyväskylä, Jyväskylä, Finland

**Keywords:** fairness, field measurements, IMU measurements, performance, safety, ski jumping, suit size

## Abstract

In the second part of this study (*Part II*), the purpose was to investigate the influence of suit size on ski jump performance during field experiments. Eight elite ski jumpers from three different nations participated by jumping in training sessions. Three different suits were made for each athlete: Suit 1 was a reference suit with current regulations, i.e., +4 cm larger than the circumference of the body, and the two other suits were respectively 2 cm larger and 2 cm smaller with respect to Suit 1. A total of 133 ski jumps were collected, with the main analysis consisting of 109 jumps as wind measurements were missing for 24 jumps. Suit size had a large influence on performance, with +11.5 pt per suit size (p<0.001), or 3.2 m per cm increase in the circumference of the suit. Wind conditions did not influence the outcome and the results were in line with *Part I*. It was indicated that the higher level athlete could benefit more from a larger suit after analyzing video of one of the ski jumpers. Inertial measurement unit measurements showed how similar length and even longer jumps could be achieved with lower speeds during the glide by utilizing a larger suit. This indicates that a larger suit could increase safety in ski jumping. However, this needs to be considered in the light of fairness, as a larger suit is more difficult to control, thus a compromise has to be made.

## 1 Introduction

The aerodynamic forces play an important role in all high-speed sports, and equipment is one of the main ways to improve aerodynamic performance. Sport equipment varies, but most sports have a high focus on enhancing their apparel and there is no consistency in how sport clothing is regulated (1). The aerodynamic behavior of fabrics has been extensively studied and has been shown to play an important role in the performance of many sports (2–4). Ski jumping is no exception, as a high-speed sport in which aerodynamic drag and lift have a major impact on the overall result (5, 6). In ski jumping, the two types of equipment used to improve performance are skis and suit, with the latter assumed to affect the most, especially relatively to suit size (6–8). This is evident from the numerous disqualifications each year of ski jumpers wearing oversized suits (9). The size of the suit influences not only the performance but also the safety, as less speed is needed in the glide with a large suit. Given the high-risk nature of ski jumping and the critical role of equipment, the International Ski and Snowboard Federation (FIS) must implement rules that ensure both safety and fairness, preserving the sport’s integrity. However, many of the current rules are based on trial and error, highlighting the need for a more scientific approach.

To understand the influence of suit size on performance, and consequently on safety and fairness, research with high internal and external validity must be carried out (10). The effect of suit size has been investigated in wind tunnels (7, 11). Here, internal validity is maintained with a high degree of control of all variables. However, such research rarely involves athletes and does not take into account how suit size influences the performance during inrun and take-off, which again is important for the overall outcome (5, 6), hence the external validity is compromised. In contrast, a field test would keep the external validity high, whilst compromising the internal validity. For a field test, a sufficient number of participants and jumps is required to be able to generalize the findings (10). Objective quantification of sport performance, such as in ski jumping, is important to avoid weaknesses of qualitative analysis (12).

Researchers have conducted field tests and data collections from competition-like settings in various forms, most commonly by video analysis of the take-off (13–17). Such analysis enables data collection from competition or competition-like settings without influencing the athletes, maintaining high external validity. Wearable sensors, such as inertial measurement units (IMUs), have been utilized to investigate jumping performance in the field in the last decade (18–23). Differential global navigation satellite systems (dGNSS) have also occasionally been used (24–27). In this case, external validity could be somewhat compromised, with the benefit of more detailed measurements. Some investigations could also be performed based on the already published FIS data, however with limited details (28). To the best of the authors’ knowledge, none of the scarce research done on ski jumping suits has been performed in the field. One reason might be that safety can be compromised in such high-risk sport, another being that a field experiment involves stakeholders that do not necessarily want to publish their results in such a competitive habitat. One would also need a sufficient number of high-level ski jumpers and jumps to be able to generalize the findings.

In order to improve both safety and fairness of ski jumping, FIS initiated a project to investigate the effect of suits on performance. For this reason, both wind tunnel measurements with high internal validity and field tests with high external validity have been performed. In *Part I* of the study, suit size and air permeability were investigated on a mannequin in a wind tunnel and numerical simulations. The purpose of this study (*Part II*) was to investigate the influence suit size had on the overall performance in the field. To decrease the number of suits per athlete, only the main parameter; suit size was investigated, as it was expected to have a greater impact on performance than air permeability.

## 2 Materials and method

### 2.1 Subjects

This data collection included eight male ski jumpers of World Cup (WC) and Continental cup (COC) level from Norway, Germany and Poland. This research was planned and organized together with professionals to ensure safety and was carried out during normal training sessions. All subjects were informed of the purpose of the study and the right to withdraw at any time prior to the test. All provided their written consent to participate and the study was carried out in accordance with the Declaration of Helsinki (29). Two of the challenges of every field test are to ensure high repeatability and a sufficiently large sample set. Hence, the aim was to recruit ski jumpers from WC and otherwise COC level with a stable performance level. Only male participants were used due to the difficulty in finding a large group of female athletes with a consistent level. The women also have slightly different rules with respect to suit size and construction (30), and a larger number of male participants was prioritized to ensure a large sample set.

### 2.2 Test protocol

The data collection was carried out by three separate national teams from Norway, Germany, and Poland, and the test protocol was agreed upon prior to the test. A total of 24 suits was made for this investigation, three for each participant. Data were collected during regular training sessions, during which athletes had to use three different suits in randomized order. A reference suit was defined after the current FIS equipment regulations (30), hereby denoted as Suit 1, and was 4 cm larger than the circumference of the ski jumper. The two other suit had a circumference of ±2 cm relative to the reference suit, denoted Suit 2 and 3 where Suit 2 was the smaller suit (+2 cm tolerance) and Suit 3 the larger suit (+6 cm tolerance) similarly as in *Part I*. The ski jumpers were instructed to jump with all three suits during the same training session. The test could not be considered blind, as ski jumpers easily feel the size difference. However, the randomized order of the suits aimed to counteract possible changes in wind during one session and a performance change during each session. All data were collected in large hills as this is the most used hill size in WC competitions. Summative information regarding data collection is provided in Table 1.

**Table 1:** Summative information on hills, hill size (HS), number of training sessions, ski jumpers performance level (WC: World Cup, COC: Continental Cup) and number of jumps in the different suits for the three teams in this data collection.

Team	Hill	HS [m]	Sessions [#]	Ski jumpers [#]		Jumps [#]		
				WC	COC	Suit 1	Suit 2	Suit 3
Norway	Granaasen	138	4	5	0	37	27	27
Germany	Obersdorf	137	3	0	1	6	6	6
Poland	Zakopane	140	2	1	0	4	4	4
	Wisla	134	2	0	1	4	4	4
Total	4		11	6	2	51	41	41

Altogether, 133 ski jumps were measured with three different suit sizes, on four different large hills, over 11 different training sessions, for eight athletes. Some extra jumps were jumped with Suit 1 during the sessions, as this was their normal suit that is usually used in trainings and competitions. For the Norwegian and German teams, wind and jump length were measured using the standard FIS competition measurement methodology (31). Together with the knowledge of the starting gate, this enabled these data to be presented in terms of performance point as in a regular competition, excluding style points. However, the Polish data did not include wind measurements, only subjective information from coaches. Hence, these data were excluded from the main analysis, but will be presented in supplementary material and used in the discussion of the results. By excluding these data, the main analysis still consists of 109 jumps (43, 33 and 33 for Suit 1, Suit 2 and Suit 3, respectively). The German team carried out additional analyses to be able to make an objective evaluation of the quality of the jumps. Here, a video analysis was carried out for all 18 jumps to record the kinematics, i.e., the body and ski posture, in relation to the trajectory. The IMU sensors used to measure the glide velocity were used for 12 of the jumps.

### 2.3 Suit construction

**Figure 1:**
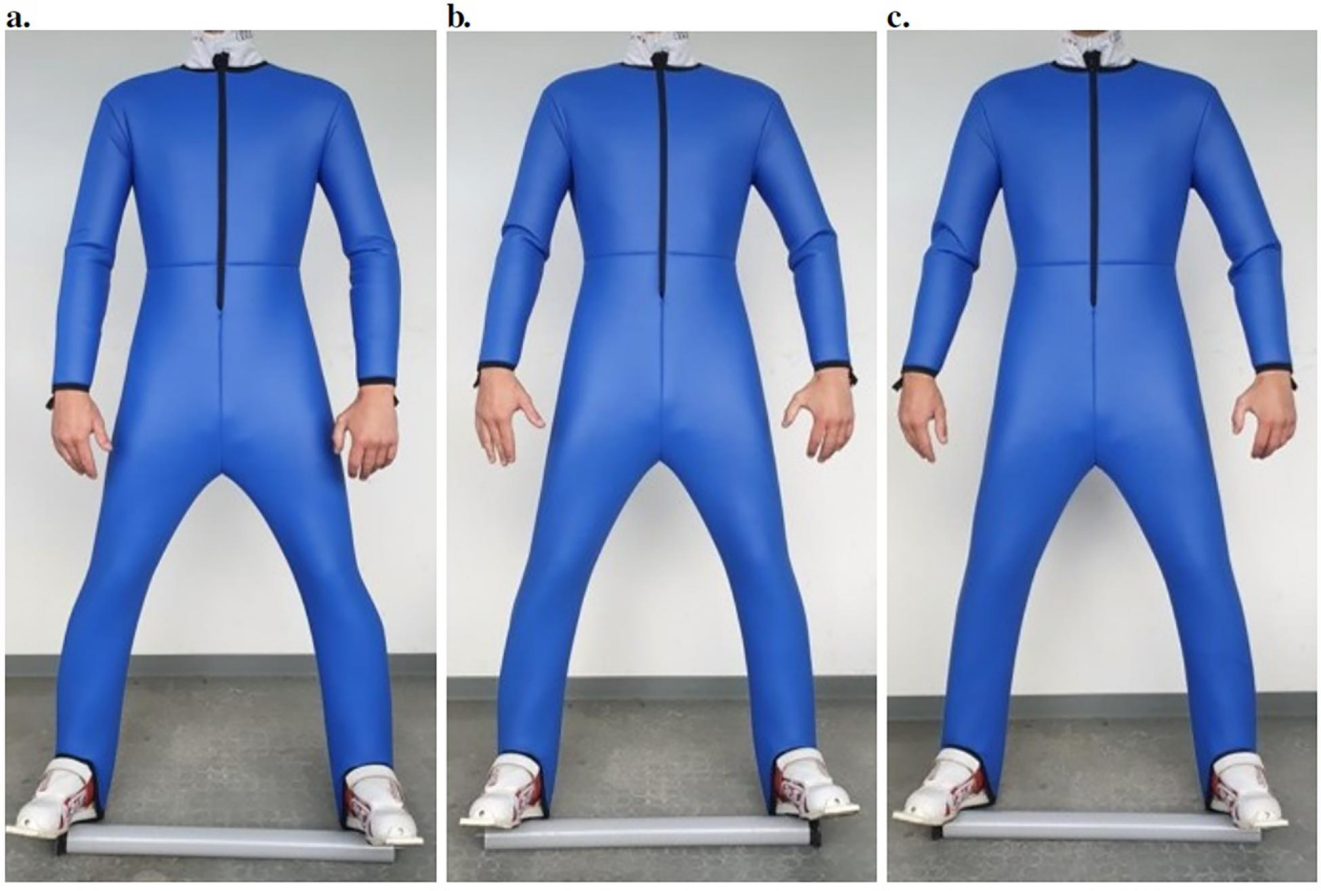
Pictures of the suits on one of the test subjects. **a** shows Suit 2 (+2 cm tolerance), **b** shows reference Suit 1 (+4 cm tolerance) and **c** shows Suit 3 (+6 cm tolerance).

The rules and regulations for the construction of a ski jumping suit are extensive (30), yet they allow for individual adjustments. Each elite athlete uses his/her own personal cut and changes could affect both the performance and safety. As the aim of this study was to investigate the change in suit size only, i.e., not the cut of the suit, each team had their own suit maker construct the suits. The suit makers and the research team met to explain how to change the suit size before construction to ensure that all teams made the same changes. The circumference of the suits was modified by changing the outside of the suit from the legs to the arms by ±2 cm from the reference suit. Example pictures of the three different suits on one of the athletes can be seen in Figure 1.

The crotch length and seams on the inside of the legs remained the same. The size around the boots was also untouched from the reference suit, as coaches and suit makers reported that this could affect safety during the take-off and glide preparation. All suits were constructed with the same material, also used in *Part I*, with air permeability ∼40 L s m^−2^. The construction of the suits and the changes made between them were in accordance with the wind tunnel test in *Part I* and a graphical illustration of how the suit sizes were changed can be found there.

### 2.4 Video and IMU analysis

**Figure 2:**
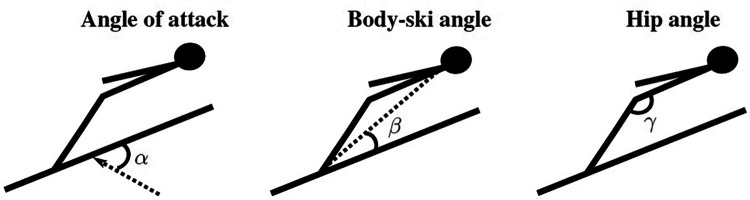
Visual description of the angle of attack (α), body-ski angle (β) and hip angle (γ) used to describe the ski jumpers posture in a similar manner as in *Part I* and literature (15, 32).

In order to evaluate the quality of the jumps, kinematic analyses of the aerial performance over the entire trajectory were carried out of the German athlete. Seven axis-cameras (50 Hz) mounted orthogonally to the trajectory were used for this analysis. For each glide section, six to eight images were analyzed using special software (“mess2d”). The resulting body and ski angles to the trajectory allow statements and comparisons to be made about the aerodynamic quality. Figure 2 displays the measured parameters.

A system with IMUs [MSR Solutions, Wangen/Allgäu, Germany (23)] was used to record the vertical and horizontal velocity (accelerometer: ±8 G, gyroscope: ±2000 ° cm^−1^, magnetometer ±8 G). This was specially developed for use in ski jumping and differs from other commercial systems. An IMU is used as a chip, manufactured using micro-electromechanical system (MEMS) technology. Each sensor was attached to the left and right ski about 10 cm in front of the binding using special double-sided adhesive pads. Calibration was carried out in a horizontal zero position (tolerance <0.05°) using a calibration table. The actual triggering of the measurement took place on the jumping hill by passing through an infrared light barrier 2 m before take-off. The measurement frequency was 500 Hz. The subsequent measurement duration was 7 s. Data were saved on a flash memory within the sensor system and then transferred to a computer via Bluetooth. This allowed to record and subsequently evaluate the entire trajectory and the associated velocities.

### 2.5 Statistical analysis

The main analysis was performed on data from the Norwegian and German teams, as wind measurements were missing from the Polish team, greatly influencing the overall outcome (33). Suit 1 (+4 cm tolerance) was defined as the reference suit, as it followed the current rules and regulations. A standard gate was set and the performance was calculated in performance points as in an FIS competition (length ± wind ± gate compensation) (34). As the suit effect could be subject dependent, the average performance of the reference suit was calculated separately for each athlete. Hence, each jump was presented as a normalized point score (Norm.point) which was the point change from the average score with the reference suit. A combination of box plots and violin plot were used for descriptive statistics using Matlab R2024b (The MathWorks, Inc., Natick, MA, USA), giving information about the median, data distribution, and density of the data. To compare the performance of the three different suits, a linear mixed-effect regression model with Bonferroni corrections was conducted. Since individual performance levels varied, suit size was included as a fixed effect, athlete as a random intercept and wind as a covariate in IBM SPSS Statistics 2017 (IBM Corp., Armonk, NY, USA). In addition, a single factor ANOVA with Bonferroni corrections was applied to investigate possible variations in wind conditions between the three suits. Alpha was set to 0.05 for all test.

## 3 Results

### 3.1 Overview

The main analysis consisted of 109 jumps from the Norwegian and German team. The average data on gate, inrun speed, jump length, wind, total points and Norm.point can be found in Table 2. Polish data are presented in Supplementary Table S1.

**Table 2:** Data (mean ± standard deviation) on speed, gate, length, wind, points and Norm.point for the three suits with different tolerance (Tol.), separated into the test team Norway (NOR) and Germany (GER).

Team	Suit	Tol.	Jumps	Gate	Speed	Length	Wind	Points	Norm.point
	[#]	[cm]	[#]	[#]	[m s^−1^]	[m]	[m s^−1^]	[pt]	[pt]
NOR	1	+4	37	6.9±3.1	24.3±0.3	133.1±6.1	1.7±0.9	74.3±11.7	0.0±11.7
	2	+2	27	8.1±2.8	24.3±0.3	129.7±6.8	1.7±0.6	62.8±12.2	−11.4±12.2
	3	+6	27	5.3±2.8	24.1±0.3	137.7±4.8	1.7±0.6	87.6±10.6	13.3±10.6
GER	1	+4	6	23.7±0.8	25.8±0.2	126.3±7.3	0.4±0.9	68.7±11.1	0.0±11.1
	2	+2	6	24.0±0.0	25.8±0.2	124.7±6.3	0.5±0.7	63.1±6.4	−5.6±6.4
	3	+6	6	23.7±0.8	25.8±0.2	130.2±6.1	0.2±0.5	78.3±10.1	9.6±10.1

**Figure 3:**
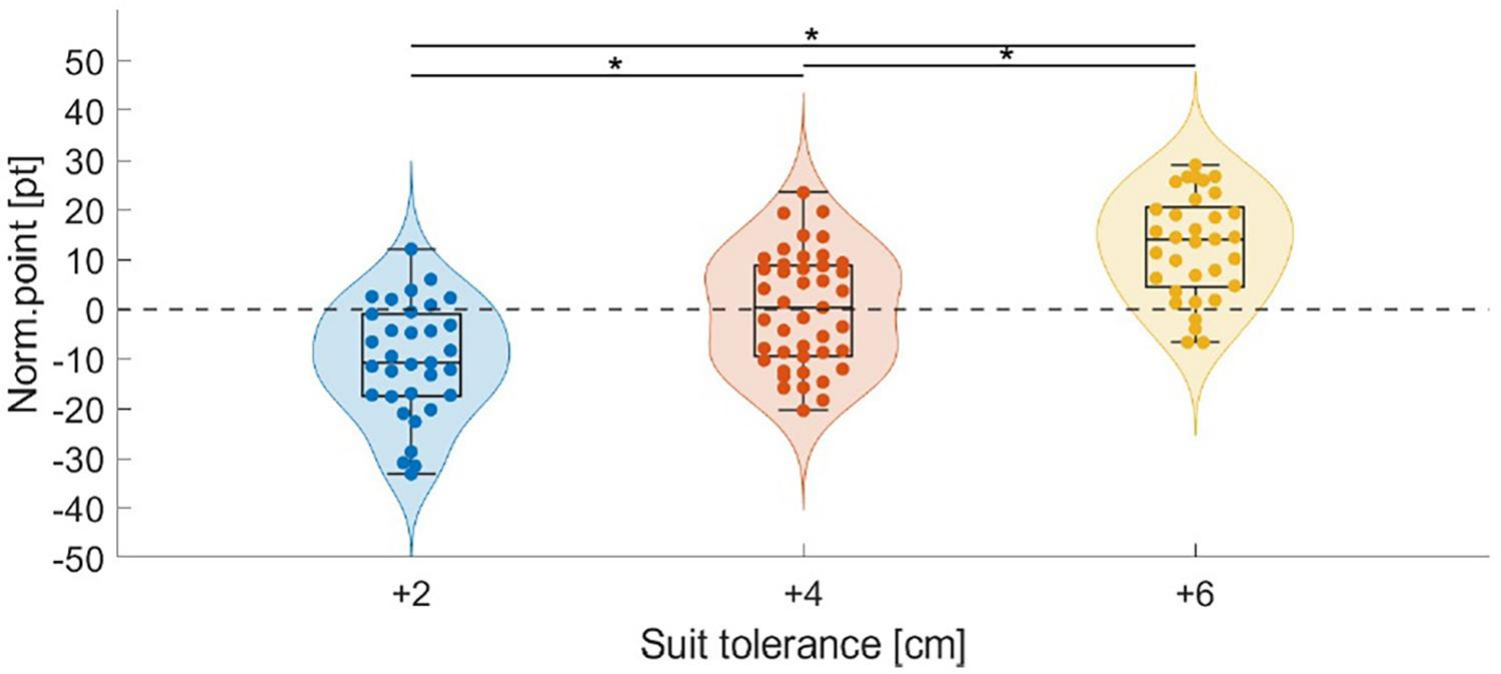
Combined violin, box and scatter plot for the combined data of the Norwegian and German team of the point change from the average score with the reference suit (Norm.point), hence the individual point difference from the average of the reference suit +4 cm. Significant difference (p<0.001) between the groups is indicated by ∗.

Overall, the ski jumpers increased their jump length by 3.8 m meters per suit, resulting in a significant point increase of 11.5 pt (p<0.001) per suit size. The jump length accounts for only 6.8 pt (meter value on large hills is 1.8 pt/m). The remaining points are attributed to gate compensation, as it was necessary to lower the gates for larger suits due to safety concerns. Possible changes in wind conditions between the suits were investigated, both for the entire group and at the individual level. There were no statistical differences in wind conditions on any level. The Norwegian team jumped in a steady headwind (+1.7 m s^−1^) which in points corresponded to -18.6 pt. The German athlete jumped in still conditions (+0.4 m s^−1^/-3.5 pt) and had somewhat more difficult conditions for Suit +6 cm, however not significant. The Norwegian team increased their average jump length by 4.0 m per suit size, resulting in an average increase of 12.4 pt. In comparison, the German athlete achieved an increase in average jump length of 2.7 m meters per suit size, resulting in a 7.6 pt increase. The combined Norm.point from both teams are displayed in a violin plot in Figure 3.

A significant difference in performance was found between all suit sizes. According to the mixed-effects regression model, the estimated mean normalized score (i.e., adjusted for differences between individual athletes and wind variability) for Suit 1 (+4 cm) was 0.0±3.4 (mean±95 % confidence interval), which was expected from the definition of the score. Suit 2 (+2 cm) scored 10.4±3.8 fewer points, and Suit 3 (+6 cm) scored 12.6±3.9 pt more than the reference suit. On average, the point scores increased with 11.5 pt per suit, hence 5.8 pt or 3.2 m per cm tolerance. The variation, density, and distribution of data were similar for all of the suit sizes.

### 3.2 Video and IMU analysis

**Figure 4:**
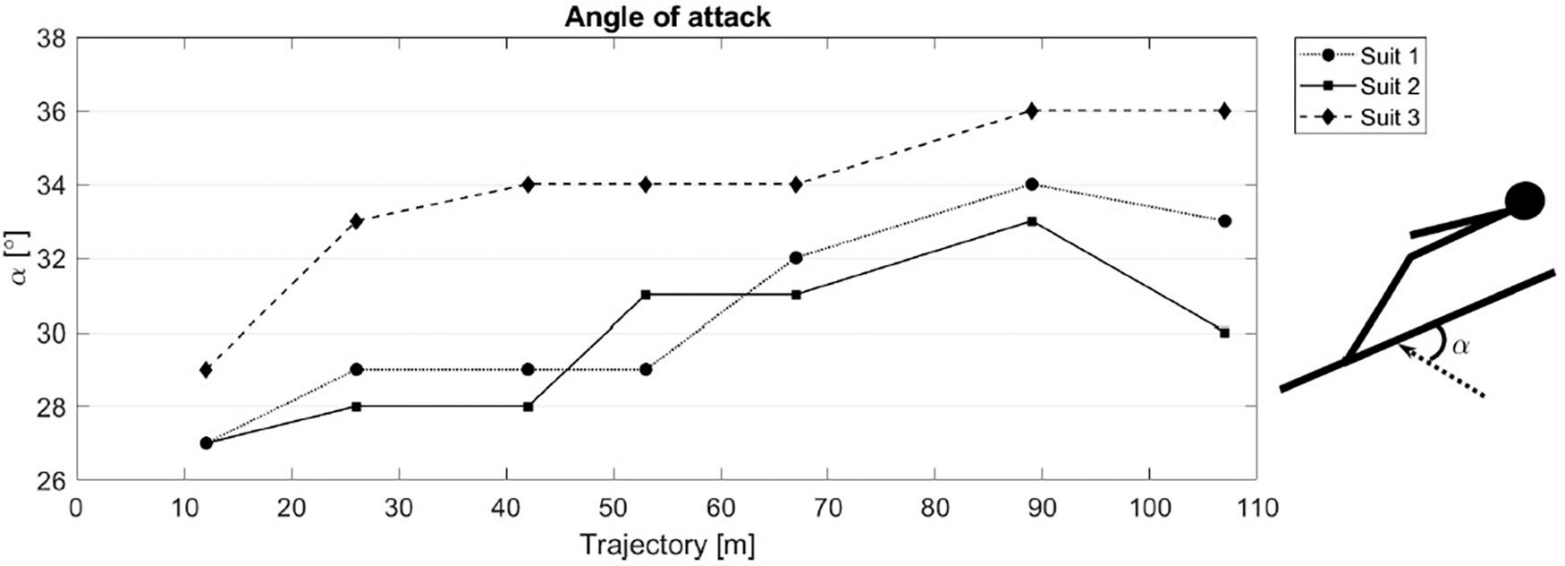
Average angle of attack of all jumps respectively carried out by the German athlete with Suit 1, Suit 2, and Suit 3.

The average angle of attack (α) is also shown in Figure 4, where the main technical differences could be observed. The complete kinetic analyses can be found in the supplementary Figure S1.

**Figure 5:**
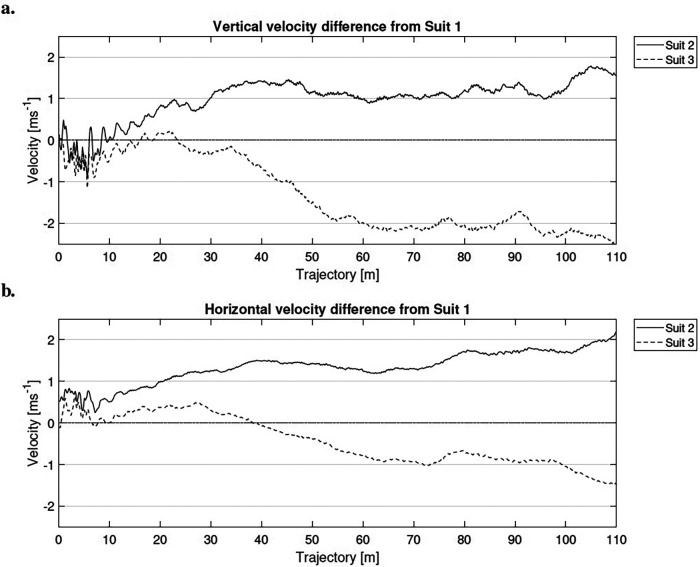
Velocity difference from Suit 1. The development of the vertical velocity is shown in **a** and the horizontal velocity in **b**.

The video analysis displayed similar angle of attack development for Suit 1 and Suit 2. With Suit 3, the athlete achieved larger angles of attack, indicating less rotation in the region 0 m to 60 m. The additional performance analysis with IMU sensors was only possible in four of the six jumps with each suit and the average vertical and horizontal velocity of the jumps are presented in Figure 5a as a velocity difference from Suit 1 (reference suit).

The ski jumper jumped ∼2 m longer with Suit 1, compared to Suit 2, even with somewhat worse wind conditions. The highest performance was observed with Suit 3, where the athlete jumped ∼4 m longer under slightly more difficult wind conditions, even without being able to achieve similar angle of attack and velocity in the first part of the glide. The highest velocity was reached with Suit 2, both vertically and horizontally. The velocity differences between the suits stabilizes from 60 m to the landing. The ski jumper maintains a vertical velocity difference of ∼1 m s^−1^ from ∼30 m with Suit 2, indicating a faster downward motion compared to the reference suit. The horizontal velocity difference continues to increase from ∼1 m s^−1^ to ∼2 m s^−1^ at the end of the glide. Hence, the highest velocities are achieved with the smallest suit, where the air resistance is assumed to be lower due to the reduction in surface area. The biggest velocity differences from the reference Suit 1 are observed for Suit 3. The ski jumper’s vertical velocity decrease more rapidly (indicating slower descent compared to the reference Suit 1) by ∼2 m s^−1^ from 30 m to 60 m and is maintained thereafter. A similar, but smaller, trend is observed for the horizontal velocity difference, which decreases by ∼1 m s^−1^, indicating reduced forward velocity compared to the reference suit. These results are directly related to the kinematic results for body-ski posture. A greater angle of attack induces a reduction in horizontal velocity.

## 4 Discussion

The purpose of this study was to examine how the most critical parameter, suit size, affects overall performance in ski jumping by using field experiments. The main finding was that increasing the suit circumference with +2 cm significantly improved the performance. Suit 1 (+4 cm larger than the circumference of the body) was used as the reference condition. Compared to this, Suit 2 (+2 cm) decreased the total point score of 10.4 pt and Suit 3 (+6 cm) increased the total point score by 12.6 pt. Hence, an average of 11.5 pt, which converts to 6.4 m (3.2 m per cm tolerance) in a large hill (1.8 pt/m). The overall result correspond well with the numerical simulations from *Part I* where the average difference between the suits were 5.6 m (10.1 pt). A slightly higher result in the field could come from an small miscalculation in the compensation system from either wind or gate, or the athlete’s technique variations. Additionally, the wind tunnel test and numerical simulations focused only on the glide phase and excluding variations in the inrun and take-off that may occur in infield data collection.

The ski jumpers had on average similar wind conditions for the different suits and within the same training session. However, the wind compensation system is complex and does for example not take cross-wind into account (33), which could have influenced the results somewhat. The numerical simulations in *Part I* showed a small benefit of having head wind as the difference between the suits increased with ∼0.2 m for 1 m s^−1^. However, such a small change would be difficult to observe in the field. The data variation were similar for all three suits, indicating that the consistency of a ski jumper is not influenced by the suit size. The total variation in the data was large, as expected from such field test. Suit size is considered an extremely important performance factor (6–8). Nevertheless, there are cases where athletes even performed better with the smallest suit, and worse with the largest one. Here, parameters such as wind conditions and gate could also play a role, as well as athlete-specific factors such as skill level and individual technique, which may limit the ability to generate sufficient rotation when aerodynamic drag increases with larger suits. However, this highlights the importance of having a sufficient number of participants, of the highest possible performance level, as well as a sufficient number of jumps when a field test is conducted (10). Increasing the number of jumping hills would also have improved the overall study, but this was unfortunately not practically possible.

The Norwegian team had in general a greater performance improvement than the German ski jumper, as well as the Polish team (NOR: 12.6, GER: 7.6 and POL: 5.9). The Norwegian team jumped both at lower altitude and with better wind conditions (stronger head wind) than the German and Polish teams, and an early hypothesis was that this could have played a role. However, the numerical simulations from *Part I* indicates that this have had limited effect on the results, around 0.7-0.9 pt. It is also difficult to interpret the Polish team without accurate wind measurements. Only looking at jump distance, the Norwegian, German and Polish athletes increased jump length of roughly 4 m, 3 m and 2 m per suit size, respectively. However, often with lower gates for the larger suits, especially with Suit 3. Video analysis was not performed for the Norwegian or Polish team. However, the German video analysis may indicate why the Norwegian team gained more in changing suits. All Norwegian athletes had frequently results among the top 15 of the World Cup during the last two seasons, whereas the German athlete competed at a lower level (COC). The two Polish jumpers represented different performance levels: one with World Cup experience and the other competing primarily at the COC level. Jumpers with qualitatively better take-off technique and movement execution in the transition phase are more likely to quickly realize enough rotation into a body posture with optimal angles of attack even with the larger suits, which could enable them to better utilize a larger suit. A plausable explanation of the superior performance enhancement for the Norwegian team. However, since all national groups performed their jumps on different hills and under varying conditions, direct cross-national comparisons should be interpreted with caution. Additional video or inertial sensor data were only available for the German athlete and might have provided further insight into dynamics and velocity profiles across athletes.

The German ski jumper had a lower rotation in the first part of the glide with Suit 3, resulting in a higher angle of attack throughout the jump. In a large hill, a higher angle of attack is associated a jump of lower aerodynamic quality (25, 35). Nevertheless, the athlete achieved the best performance with Suit 3, suggesting that the increased aerodynamic parameters from the larger suit may have compensated for the suboptimal posture. A clear limitation with the IMU analysis was that the investigation only included one of the eight athlete, jumping in only one of the included hills. However, the speed analysis corresponds well with the findings from *Part I*. The aerodynamic forces increase with suit size, which reduces the velocity, especially in vertical direction. This confirms expectations regarding the influence of suit size and the importance suit size also has for safety in ski jumping. This is a clear indication of the advantageous influence of larger suits on the achievement of long jumping distances. However, it also shows that this advantage goes hand in hand with the athlete technique.

Altogether, this two-part study has shown the importance of suit size. Whilst the wind tunnel test and numerical simulation (*Part I*) showed that the jump length theoretically increased with 2.8 m per cm tolerance, the field test (*Part II*) showed a average increase of 3.2 m, hence in good agreement. Air permeability did not have a large effect in *Part I*, but should also be tested in the field in future research. It is clear from this study that similar and larger jump length could be reached with reduced speed by wearing a larger suit. Especially the reduction in the vertical velocity is considered important for the safety of the ski jumpers with respect to the landings. However, the disadvantages of a larger suit is the fairness consideration of the sport. A larger suit can be difficult to control and the role of the equipment could overshadow the physical performance. This must be considered by FIS, which needs to find a good compromise. For future research, equipment changes that could improve both safety and fairness should be investigated.

## 5 Conclusion

In two parts, this study has investigated the influence suit size has on ski jumping performance. Here in *Part II*, an extensive field test was performed that involved elite athletes of three different nations. Suit size was shown to be an important performance factor, where the performance increased significantly with 5.8 pt/cm in suit circumference. This translates into a 3.2 m increase in jump length per centimeter of tolerance, which is well aligned with the findings in *Part I*. The athlete showed similar variation in performance for the different suit sizes while the wind did not affect the results. By increasing suit size, a ski jumper can reach a similar, or even longer, jump length with less speed, which is beneficial for enhancing safety of the sport. However, the performance gains from larger suits may depend on the jumper’s technical ability to maintain optimal body-ski posture and control. Furthermore, larger suits raise concerns about fairness in competition, as equipment advantages might overshadow physical performance. Future research should explore equipment innovations that enhance both safety and fairness, possibly supported by comprehensive biomechanical assessments and wind-controlled testing environments.

## Data Availability

The raw data supporting the conclusions of this article will be made available by the authors, without undue reservation.
