# Supplementary material for: The influence of suit size on performance in ski jumping. Part II: field measurements
Source: Front Sports Act Living. 2026 Feb 13;8:1693723. doi: 10.3389/fspor.2026.1693723 (PMC12945395; doi:10.3389/fspor.2026.1693723)
Supplement: Supplementary file 1 [file Datasheet1.pdf]

Supplementary Material

1 DATA FROM THE POLISH TEAM

Table S1 displays an overview over the data from the Polish team.

Table S1. Data (mean±SD) on speed, gate, jump length, total points and Norm.point for the three different suits for the Polish team.

Nation	Suit [#]	Tolerance [cm]	Jumps [#]	Gate [#]	Speed [$\mathrm{m\,s^{-1}}$]	Length [m]	Points [pt]	Norm.point [pt]
Poland	1	+4	8	19.5±1.8	25.5±0.2	127.9±11.0	66.7±8.9	0.0±5.4
	2	+2	8	19.5±1.8	25.5±0.2	122.9±9.3	57.7±5.2	-9.0±3.2
	3	+6	8	18.5±2.8	25.4±0.1	126.8±7.4	69.5±7.3	2.8±3.9

The data was not used in the general investigation as exact wind measurements were missing. However, subjective wind was reported by the coach of the athletes. Similarly for all suits, six of eight jumps were reported to have $0.5\,\mathrm{m\,s^{-1}}$ to $1.0\,\mathrm{m\,s^{-1}}$ tail wind, one jump without wind and one jump with $0.5\,\mathrm{m\,s^{-1}}$ to $1.0\,\mathrm{m\,s^{-1}}$ head wind.

2 MEASURED BODY ANGLES

Figure S1 displays the kinematic analyses of the flight posture of the German athlete, described in terms of angle of attack, ski-body angle and hip angle consistent with *Part I* and recent literature.

a.

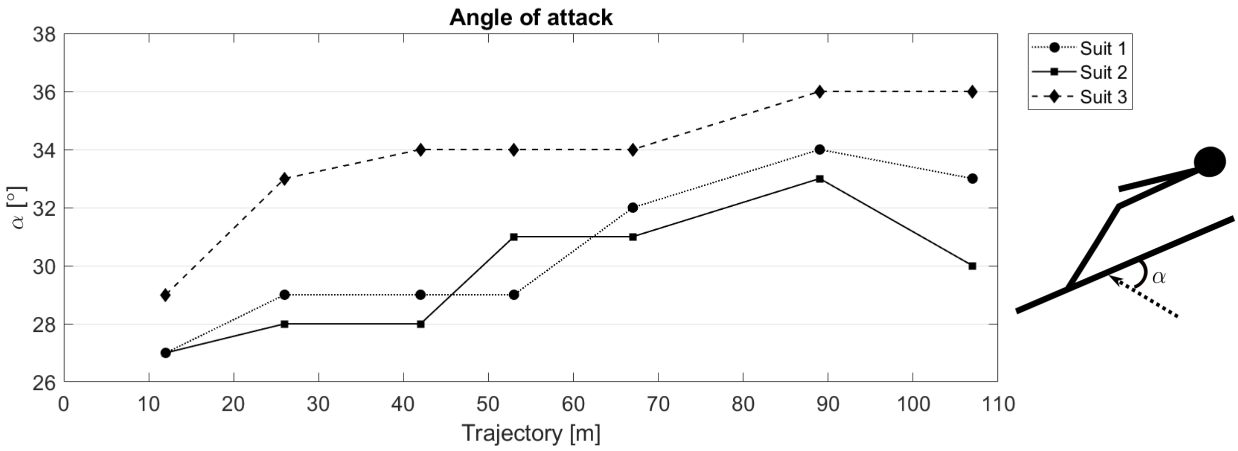

b.

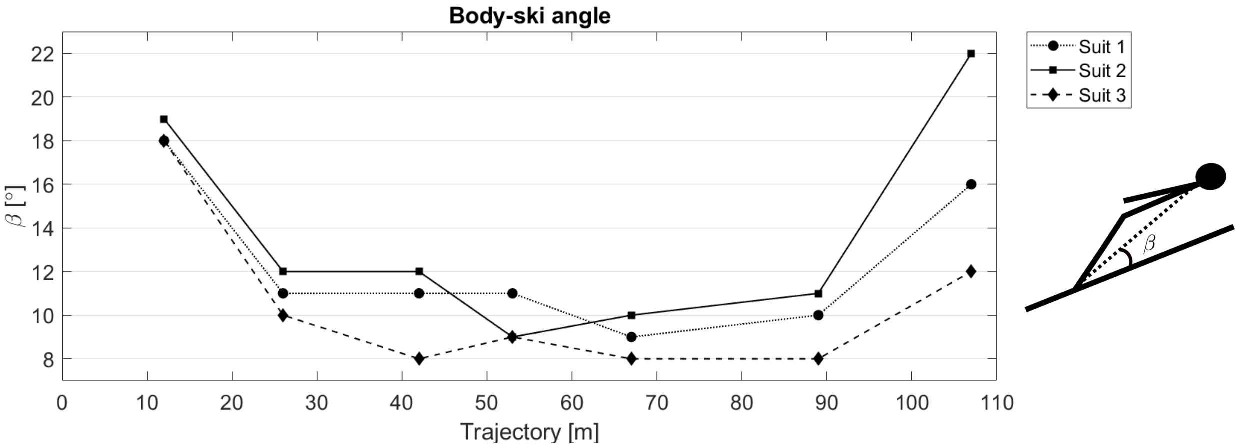

c.

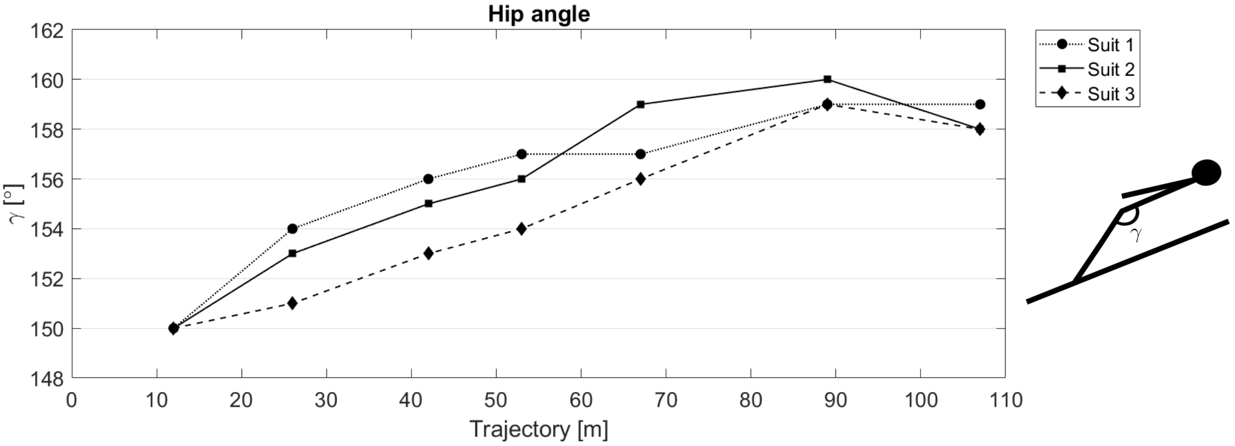

Figure S1. Average measurements of angle of attack (α) in **a**, body-ski angle (β) in **b** and hip angle (γ) in **c**, for the German ski jumper with the three different suits.
